# Understanding Dietary Diversity, Dietary Practices and Changes in Food Patterns in Marginalised Societies in Sri Lanka

**DOI:** 10.3390/foods9111659

**Published:** 2020-11-13

**Authors:** Permani C. Weerasekara, Chandana R. Withanachchi, G. A. S. Ginigaddara, Angelika Ploeger

**Affiliations:** 1 Specialized Partnership in Sustainable Food Systems and Food Sovereignty, Faculty of Organic Agricultural Sciences, University of Kassel, 37213 Witzenhausen, Germany; a.ploeger@t-online.de; 2 Department of Archaeology and Heritage Management, Faculty of Social Sciences and Humanities, Rajarata University of Sri Lanka, Anuradhapura 50000, Sri Lanka; chandanawithanachchi@gmail.com; 3 Department of Agricultural Systems, Faculty of Agriculture, Rajarata University of Sri Lanka, Anuradhapura 50000, Sri Lanka; sanjeewanieg@gmail.com

**Keywords:** dietary diversity, food insecurity, traditional food pattern changes, nutrition and health, food practices, women of reproductive age, marginalised areas

## Abstract

Micronutrient malnutrition is a serious public health problem in developing countries, including Sri Lanka. Most frequently, micronutrient malnutrition is experienced by the poorest households due to cereal-based, monotonous diets that lack dietary diversity. Sri Lankan traditional food system is changing day by day. In parallel, nutrition deficiencies, malnutrition, and noncommunicable diseases are the most significant problems today in Sri Lanka. Therefore, understanding dietary diversity and dietary changes in Sri Lanka must be studied to address related public health issues. This study investigates nutrition adequacy, dietary diversity, dietary practice, and traditional food pattern changes in different marginalised areas in Sri Lanka. A cross-sectional survey was done using 24 h food recall and an administrative questionnaire for 400 women of reproductive age (WRA) (18–49 years old) in marginalised areas in Sri Lanka. The random sampling method was used for data collection. The research confirmed that different areas had poor dietary practices, with macronutrient imbalance and alarmingly low intakes of micronutrients. Interestingly, the diversity of food was higher in rural areas than in urban areas. Approximately 83% of women in urban areas did not meet minimum dietary diversity (MDD-W). Overall, about 63% of reproductive age women did not meet MDD-W and food security. The mean MDD-W for both areas was low. Significant differences in MDD-W levels were found in the two areas (F = 90.483, *p* < 0.05). The sample showed a significant positive correlation between MDD-W and area, monthly income, educational level, food source, BMI level and health status (*R*^2^ = 360; *p* < 0.01). This sample did not find that the consumption of traditional varieties of foods and agrobiodiversity are decreasing. Additionally, the study confirmed that low dietary diversity impacts nutrition status and health. The results highlight that the leading causes for low diet diversity are decreased dependence on own production, increased purchasing food at markets, lack of suitable lands to cultivate, agro-commercialisation, less knowledge of food and nutrition, loss of traditional food culture, low income and high prices of food. Inappropriate food patterns, nutrition policies and governance in Sri Lanka are the main factors to the nutrition findings. The study finding will help the decision-making authorities or policymakers to design suitable nutrition programs for vulnerable people in marginalised areas and to use these to strengthen a sustainable food and nutrition system in Sri Lanka.

## 1. Introduction

Proper nutrition is an essential aspect of a healthy lifestyle and disease prevention [1]. An inadequate micronutrient intake or lack of dietary diversity is a global challenge, and it is a direct outcome of the inability to obtain nutrient-rich, well-balanced food [2]. As a result, many people worldwide suffer from one or more forms of malnutrition [3,4,5,6]. Malnutrition exists in various forms, including micronutrition deficiencies, undernutrition, overweight, obesity and noncommunicable diseases. The reasons for not eating a healthy diverse diet are complex and multifactored. However, they include a lack of access to foods, a lack of knowledge on diverse diet composition, cultural norms, traditions, poverty, and food and nutrition governance. International and national level food policy may contribute to the erosion of local crops and varieties that underpin traditional dietary diversity [7]. Many individual crops are locally valuable but are neglected by policymaking [7,8]. Thus, Dietary Diversity (DD) is internationally recognised as a vital element of healthy diets [9] as it is a measurement of diet quality [10].

Dietary Diversity (DD) is defined as the number of different food groups or foods consumed in a given period [10]. Diverse foods are a good source for various macro and micronutrients and best ensure nutrient adequacy [10]. Increased risk of chronic diseases and malnutrition are associated with dietary factors, and international and local guidelines recommend improving dietary diversity [10]. Therefore, DD is needed to meet energy requirements and other necessary nutrients, especially for those at risk for nutritional deficiencies and chronic diseases [10]. More so, DD is a key measure of sustainable diets and is currently being considered as one of the principal sustainable diet indicators for the United Nations’ Sustainable Development Goals (SDGs) [11]. Understanding dietary diversity may be a convenient pathway to evaluate inadequate micronutrient and household-level food security [12,13,14], sustainable dietary practices and food pattern changes [12]. For those reasons, improving DD has been proposed as one approach to micronutrient deficiency and food insecurity by the Food and Agriculture Organization (FAO) [12]. Some studies have shown that dietary diversity can promote a healthy weight [15,16,17], improve nutritional status [18,19], and foster a healthier lifestyle [20,21]. It has also been shown to improve food security [15,22], and it is associated with high agrobiodiversity and high food self-sufficiency [23]. Although DD is universally recognised as a vital component of healthy foods, DD is not yet used as a dimension of diet quality [10,12], and there is a lack of consensus to operate and evaluate it [24].

Sri Lankan traditional diets have had a rich diversity [25] and offer various health and nutritional benefits, including protection from noncommunicable diseases (NCDs) and micronutrition deficiencies. Usually, these foods have been obtained from natural sources, and proteins have been available from different plant varieties. The types of traditional foods, preparations, and consumption habits were more heterogeneous than today [25]. Traditional Sri Lankan food has many nutritional benefits, and the Sri Lankan diet consists mainly of green leafy vegetables [25]. Green leaves were a major source of vitamins and other therapeutic values [25]. However, the Sri Lankan traditional food system is changing day by day.

Today, many health and nutritional problems in Sri Lanka are due to unhealthy eating patterns and low-quality food intake [9,26,27]. In parallel nutrition deficiencies, malnutrition and noncommunicable diseases are the most significant problems facing Sri Lanka [28]. The low birth rate is 17%, and one-sixth of women have low BMI levels [29]. The prevalence of anaemia is 19.6% among lactating women, 16.2% among reproductive women [30,31]. Overall, 32.6% of WRA suffer from anaemia [32]. Vitamin A deficiency has been found in around 15% of mothers with children (6–60 months) [33]. Other micronutrient deficiencies such as iodine, iron, zinc and vitamins A, D and B are reported in different age groups in Sri Lanka [34,35]. As mentioned by World Health Organization (WHO) 2018, noncommunicable diseases (NCDs) including diabetes, cancer, heart disease and chronic diseases account for 83% of all deaths in Sri Lanka [28,36]. These data illustrate the problems associated with unsustainable eating patterns and a low-quality diet in Sri Lanka. Women of reproductive age (WRA) are especially vulnerable to nutritional deficiencies, however inadequate nutrient intakes during reproductive age can affect both women and their infants [37]. With this understanding, dietary diversity and dietary changes in Sri Lanka are important to be studied. However, a systematic study of DD, nutrient adequacy, dietary practices and changes in traditional food patterns in different marginalised areas have not been conducted in Sri Lanka. Accordingly, a cross-sectional survey using an administrative questionnaire and a 24 h food recall was conducted among 400 WRA (18–49 years old) in the marginalised areas of Sri Lanka. The minimum dietary diversity of women of reproductive age (MDD-W) was used as the main measure for DD and dietary changes. Data were gathered using a random sampling method. This study seeks to understand the food transition, current sustainable dietary practices, policies and governance among the sample population. It supports evaluating micronutrient adequacy and assessing the impact of DD on nutrition and health conditions. Sub-objectives of the study are to understand the changing agrobiodiversity, food and nutrition governance, sustainable dietary practices, and micronutrition problems and food transition in rural-urban marginalised areas, and to assess household food security. This research study seeks to improve nutritional health through food systems by moving towards a more sustainable Sri Lankan food system.

## 2. Materials and Methods

### 2.1. Study Areas

The study is part of a collaborative project investigating food system commercialisation and hidden hunger and malnutrition in Sri Lanka [25,38]. Based on the feasibility and previous research experience carried out in Sri Lanka in August 2016, the agro-environmental zone for rural areas was selected for this project. This study was conducted in marginalised areas (urban slums and remote rural areas) in Sri Lanka due to socioeconomic and health-related problems. The district of Anuradhapura in the North-Central province of Sri Lanka was chosen as the remote rural area. People in this area are mainly engaged in small-scale agriculture [39]. The region has endured 30 years of war (war border villages), and inhabitants also face water scarcity, poverty, malnutrition, as well as chronic kidney diseases [39]. The urban slum and shanty area selected was the Colombo district where two divisional secretariats were selected. Residents are classified as low-income and suffer from malnutrition, social and health problems, poverty, inadequate living space, poor living conditions and high unemployment rates [39].

### 2.2. Study Sample

Ethical approval for this research project was obtained from the Ethics Review Committee (Ref: ERC/09/19) of the Faculty of Applied Sciences at Rajarata University, Sri Lanka. Each woman gave written informed consent before enrolment. A cross-sectional survey was delivered from December 2018 to March 2019 to assess the socio-demographic characteristics, health, nutrition and dietary diversity of 400 of WRA in marginalised areas of Sri Lanka. DD data were collected by administering a questionnaire survey to randomly selected households of WRA in both areas. All of the interviews were led in the Sri Lankan official language. Enumerators were trained on how to carry an interview. According to a feasibility study, women are a strong indicator in these areas; they help improve food security and suffer from nutrition-related problems and health issues while most women are the main income holders. Thus, in both areas, 400 WRA (18–49 years old) were randomly selected from the electoral division’s list of divisional secretariats by age. In contrast, 200 rural households and 200 urban households were selected for the WRA. The sample size 400 was selected, indicating a confidence gap of ±2.5% (total = 5%). One woman from each household was chosen. Where a woman refused to participate in a survey, or no woman was available from the selected household, then a WRA from the next nearest household was chosen. All women took part in this study (*n* = 400; 100%). No erroneous data were found; the Enumerators completed the questionnaires through interviews. No monetary compensation was paid to the participants.

### 2.3. Minimum Dietary Diversity of WRA (MDD-W)

The Minimum Dietary Diversity for WRA (MDD-W) can be applied as a proxy indicator of high micronutrient adequacy, one of the critical components of food quality [37,40]. Therefore, it could be used to measure household access to a micronutrients rich diet [10,37,41]. Furthermore, MDD-W is remarked as a traditionalist evaluation of micronutrient adequacy of women’s diet and household nutritional security [10,37,42]. Thus, in this study, MDD-W was used to understand the dietary diversity in these areas. DD was recognised by using the approved 24 h recall method adopted from food and agriculture organisation (FAO) [37]. Women were first asked to recall all dishes, drinks, snacks and other food they had consumed from the time they woke up the following day. All participants were encouraged to remember all food and beverages consumed within the previous 24 h. Women were also instructed to describe all of the ingredients in the mixed dishes, the source of the ingredients (purchase, collection or donation, cultivation), and the method of preparation. All ingredients were directly coded by a well-trained researcher and classified into a predefined list of 10 food groups [37]. Ten food groups were added as proposed by the FAO, MDD-W Guidelines [37] that consist of (1) all starchy food (grains, white roots, tuber and plantation); (2) beans and peas; (3) nuts and seeds; (4) dairy products (milk, yoghurt, and cheese); (5) meat products (fish, meat, poultry, liver or organ meats); (6) eggs; (7) dark green leafy vegetables; (8) vitamin A-rich fruits and vegetables; (9) other vegetables; (10) other fruits. Each group was designed with a score of 1 if consumed and score 0 if not consumed. These food groups were reviewed across 11 notable micronutrients (vitamins B12, B6, A and C, calcium, iron, zinc, thiamine, riboflavin, niacin and folate) [11] and excluded components of fats and oils because they are not considered micronutrients [37,41]. A woman was classified as having food insecure or inadequate dietary diversity if she had consumed less than five food groups. A woman who consumed five or more food groups reached MDD-W with good diet variety and food security [37,41].

### 2.4. Assessment of Food and Nutrient Intakes

Nutrient Intake was calculated and used to compare daily recommended nutrient intakes. Since MDD-W only takes micronutrients into account, calculating nutrient intake provides insight into micro- and macro-nutrient intakes [10,37]. Food intake was evaluated using a multiple pass 24 h diet recall conducted by well-trained field workers [10]. Women were asked to describe all food and beverages they had consumed during the preceding 24 h, including cooking methods, time of consumption, and portion size [37,41]. Several visual aids were used to estimate the portion sizes, including standard pots, plates, bowls, and spoons. The Sri Lankan food consumption table [43] and the Nutri-survey for Windows software [44] were used to calculate dietary intake of macronutrients (fat, protein, and carbohydrate) as well as micronutrients (iron, zinc, calcium, vitamins A, B6, B12 and C, thiamine, niacin, riboflavin and folate). The Estimated Average Requirements (EAR) as proposed by the WHO/IMPMUS [45,46] and the National Academy of Science were used to calculate the probability of adequacy for each nutrient.

### 2.5. Health Condition

In this sample, self-reported health conditions were examined. Women were asked to report their health condition for the last seven days.

### 2.6. Biodiversity and Dietary Practices and Food Patterns

According to 24 h open recall and list-based recall, we examined the diversity of food in both areas. Food pattern transition was examined by the administered questionnaires and some interviews about the traditional food.

### 2.7. Anthropometric Measurements

Anthropometric measurements were made using a stadiometer and electronic weighing scale using trained enumerators. The height was measured to the nearest centimetre. Participants were asked to wear light clothes and remove shoes [47]. The Body Mass Index (BMI) was calculated by dividing weight (kg) with height in metres squared (kg/m^2^). The nutritional status was assessed using the BMI according to the criteria of the World Health Organization [48].

### 2.8. Statistical Analysis

The data were entered using an Excel sheet, and all statistical analyses were done using SPSS statistical version 21.0 (IBM, Armonk, NY, USA). All data were double-checked to prevent errors. Demographic and socioeconomic characteristics data were presented as Mean and standard deviations and percentages. The ANOVA, *t*-test, Mann–Whitney, and Spearman’s rho (r) correlations were performed to compare variables. The MDD-W statistical differences between the two areas were tested using ANOVA and nonparametric tests. The multiple linear regression model was used to understand the associations between MDD-W and selected socioeconomic indicators. All statistics were checked using two side tests, and significance was used at *p* < 0.05, *p* < 0.001. Nutrition intake was calculated using Nutri-Survey software [44] and the Sri Lankan food consumption table [43].

## 3. Results

### 3.1. Demographic and Socioeconomic Characteristics

The sample included 400 WRA between the ages of 18 and 49 years old. The majority was between 26–35 years old and 36–49 years old (68%) and more than half had some level of education (58.5%). Most of the women sampled came from low-income households, and about 60.5% were housewives without an income. In rural areas, the main occupation was agriculture. In contrast, urban slum women occupied hard jobs such as domestic workers or labourers on road and building construction. About 50% of families spend less than USD 55 a month on food expenditures. Those without income relied on *Samurdhi* aid [49,50] from the government amounting to only USD 13 per month (Rs.1 = 0.0054 Dollars). More than half of the women suffered from nutritional problems such as overweight, obesity and underweight. In general, food sources obtained by households in both areas were purchased. However, in rural areas, more than 70% of women produced their food, 14% collected wild fruit and vegetables, and 2.5% received some donations from the government or non-government organisations (NGOs) (collected and donated 16.5%). In comparison, more than 90% of women in urban areas purchased food from different sources and did not report producing their food. In urban areas, only 1.5% also reported collecting donations from the government (see Table 1).

### 3.2. Dietary Diversity

**Figure 1:**
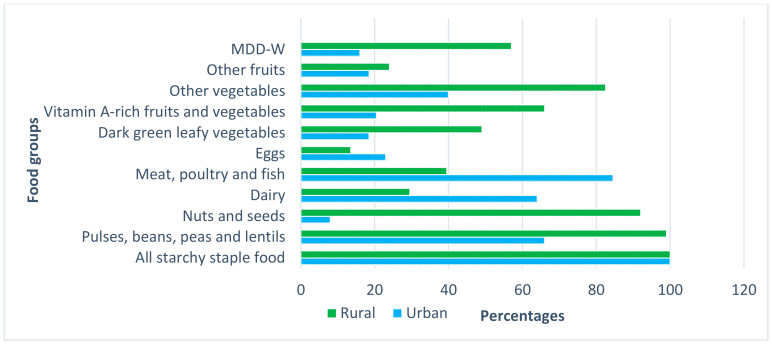
MDD-W (Minimum dietary diversity for women of reproductive age) 10 food groups of women of reproductive age in urban and rural areas.

Minimum dietary diversity for women of reproductive age (MDD-W) was calculated from 10 food groups using a 24 h recall. The first group (all starchy staple foods) were consumed by 100% of women in both areas with the highest intakes. The majority of foods consumed by women include rice, rice flour products (string hoppers, hoppers, *pittu*, noodles) and wheat flour products (bread, bun, *koththu*, noodles). More than 50% of women in urban areas consumed meat, poultry, and fish, pulses and dairy. Over 80% of women in urban areas had consumed animal sources of food in the last 24 h. In rural areas, women consumed starchy staple food, and about 90% of women consumed pulses, beans, peas and lentils (Group 2), nuts and seeds (Group 3) and other vegetables (Group 9). More than 60% of women in rural areas consumed vitamin A-rich foods, while in urban areas, women did not consume dark green leafy vegetables (Group 7) and vitamin A-rich fruits and vegetables (see Figure 1).

### 3.3. Agro-Biodiversity, Dietary Practices and Traditional Food Patterns

Diet diversity was richer in rural areas than in urban areas. There was a significant positive correlation between the areas and food groups 2 to 9 (*p* < 0.01). There was no significant correlation between areas and food group 10 (other fruits) as both areas had low consumption of fruits. Furthermore, in both areas, 100% of participants reported eating starchy staple food, but the variety of grains, tubers, white roots, and plantation foods were wider in a rural area. In the study, sample results revealed that most of the women were consuming a low percentage of animal protein. Rural women were consuming wider varieties of dark green leafy vegetables. Additionally, the rural area was higher in participants with food self-sufficiency. Nevertheless, study results revealed that women of reproductive age (WRA) were not consuming the traditional variety of foods such as healthy wild plants. Different food and drink items are listed in the following table (see Table 2).

In Sri Lankan, indigenous fruits and vegetables were popular in the past and used for a variety of health and nutrition benefits [25] (see Table 3).

The table shows some of the traditional food variety in rural areas. For example, traditional people ate “Finger millet/*Kurakkan*” daily, and it was one of the main staple foods available to them. They also used jack seeds kept under the sand as they believed this helped reduce the possible harmful effects with consumption. Most green vegetables and leaves listed contain high amounts of minerals and vitamins that play a significant role in boosting the immune system. These foods were and are still often available in the forest area or home gardens. Unfortunately, in this sample, the consumption of these foods was not found. A notable point of this study is that this population’s consumption of traditional food varieties or diversity of food has been rejected, which could be contributing to micronutrition deficiencies. However, since urban area households consume mostly bought food from the market. The results show that DD among both groups differed significantly, even when both did not report eating traditional food varieties. Our findings indicate, nonetheless, that agrobiodiversity is decreasing.

### 3.4. Minimum Dietary Diversity

The mean minimum dietary diversity for WRA was 1.37 ± 0.483. About 57.5% of women in rural areas consumed ≥5 food groups. Approximately, 16% of women in urban areas consumed ≥5 food groups. In both areas, 36.75% of women consumed >5 food groups, while 63.25% of women failed to meet the minimum food diversity and food security. Overall, the mean MDD-W for both areas was low. Significant differences were found in MDD-W among the two areas (F = 90.483, *p* < 0.05) (see Table 4).

**Figure 2:**
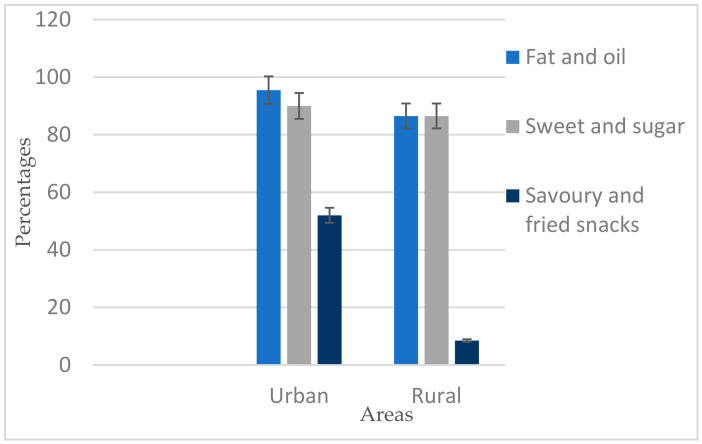
Percent consuming low nutrient density food groups.

These data showed a clear pattern of rural-urban integrity in the diets of women. The lowest MDD-W was seen in urban areas. On the other hand, this study examines the low percentage of WRA consuming foods from specific food groups and subgroups and thus provides a good qualitative description of the diet. The study found that WRA were consuming a high percentage of low-nutrient density food groups (see Figure 2).

WRA consumed more than 80% of fat, oil and sweets in both areas, while more than 50% of urban women consumed savoury (salty, pleasant, appetising) and fried snacks. There were no significant differences in both areas (*p* > 0.001).

### 3.5. Associations between Minimum Dietary Diversity for WRA (MDD-W) and Socioeconomic Indicators

The sample showed a significant positive correlation between the minimum dietary diversity of WRA and area, monthly income, level of education, food source, BMI level, and health status (correlation is significant at 0.001). There was no significant correlation between minimum dietary diversity of WRA and age, food expenses, household size, marital status, and main occupation (see Table 5).

In the multiple regression models, there was a significant difference between the monthly income, area of study, education level, source of food, BMI level and health status of reproductive age women (*R*^2^ = 360; *p* < 0.01). Results showed the rural people had more dietary diversity than urban people. Women with a higher level of education had better dietary diversity than women with a lower level of education. In addition, women with good nutrition status had varied diets. Women who had high-income had more dietary diversity than lower-income women. Additionally, women who consumed their own production had better dietary diversity than other women.

The results showed a significant positive correlation between MDD-W and BMI status. Study results showed that underweight women had low dietary diversity, and women who had an average weight had minimum dietary diversity. The study results revealed that health conditions such as heart diseases, diabetes, cancer, kidney diseases, high blood pressure and eyesight problems were prevalent in reproductive-age women in the study area. Spearman’s rho (r) correlation was examined to confirm the relationship between the MDD-W and the health status of the study sample. There was a significant positive correlation between MDD-W and the health status of the study sample.

### 3.6. Respondents Health Conditions

In this study, the self-reported health condition was investigated. About 30% of the participants reported having experienced bad health status, while 60% reported being healthy. The study results suggest that conditions such as heart disease, diabetes, and high blood pressure are more common in urban women than in rural women. Additionally, the study results showed that other diseases such as being overweight or obese, tooth decay, high cholesterol, stroke, depression, anaemia, eating disorders and swollen legs were more common in urban women than in rural women (see Table 6).

### 3.7. Nutrition Intake and Nutrients Adequacy of Women

According to Nutri-Survey software and Sri Lankan food consumption table data, the mean energy intake was 1.04 ± 0.196. There is no statistically significant difference in macronutrient intake for reproductive women between urban areas and rural areas. Overall, the mean energy consumption was 2230 kcal/day, with 80% coming from carbohydrates, 9% from proteins and 11% from fat. While 96% of women met the daily recommended carbohydrate intake, only 27% met daily recommended protein intake. In this sample, it was found that women in both areas did not meet the minimum daily micronutrient intake recommendations. Less than the mean EAR for eight micronutrients, but not for thiamine, niacin, and riboflavin. Additionally, the consumption of water was low among reproductive-age women in these areas. This data indicates that the study sample had a high risk of micronutrient deficiencies for example, for vitamin A, vitamin C, calcium and low levels of other nutrients crucial for reproductive-age women such as iron, vitamin B and protein (see Table 7).

## 4. Discussion

The WRA for minimum dietary diversity (MDD-W) is a flexible tool that can be used to compare and categorise women’s dietary diversity within and across different settings [37]. Due to the emerging use of MDD-W, few studies have reported MDD-W prevalence values [41,42,57]. A developing country, such as Sri Lanka, is undergoing a rapid epidemiological and nutrition transition [25] attributed to low levels of food and nutrition-related knowledge [38]. These food patterns are associated with an increased risk of micronutrient deficiencies and health problems [40,58]. Additionally, a monotonous diet along with household food insecurity [59,60,61] are most frequently experienced by the poorest households [61,62,63].

Sri Lanka has a rich and wide variety of edible food species [64]. According to our recent study, Sri Lanka had a rich dietary diversity in the past [25]. Yet today, urbanisation, food preferences and lifestyles have led to changes in food production, eating habits and food systems leading to growing health and nutritional problems. Food variety and dietary diversity scores are positively related to socioeconomic factors [14,19]. Therefore, understanding dietary diversity may be a more accessible pathway to evaluating household-level food security [13]. It can further help understand health and nutrition status, food, agrobiodiversity, healthier lifestyle, food transition and micronutrient adequacy [24,65]. Our study results helped to understand food security, nutrition and diversity of food, sustainable dietary practices and the overall health situation in marginalised areas of Sri Lanka. It allows us to differentiate women at different levels of vulnerability [40]. Furthermore, it deepens the understanding of the diversity of diets and the agrobiodiversity of households.

### 4.1. Dietary Diversity and Micronutrients Adequacy

In the study sample, MDD-W was classified as less than five groups of foods consumed by women. This research outcome increases their vulnerability to food and nutrition insecurity. Previous studies found the lowest levels of dietary diversity in different areas among Sri Lankan populations [26,66,67]. However, no research was found considering female reproductive age besides this investigation in marginalised societies. Compared with other Asian countries, Sri Lanka records a higher percentage of MDD [68]. In the rural study area, some endemic vegetables and fruits may be helping to achieve food security. Common vegetables and fruit in these areas have a variety of nutritional benefits that include leafy greens consumed as part of their regular diets and used in various ways [25].

Since most of the households partake in small scale agriculture, they are provided with better food security. Some of their food proportions, especially vegetable legumes, different kinds of mushrooms, nuts, seeds and leafy greens from the wild, are considerably higher than in urban areas. Wild foods can support households who experience financial difficulties and are important contributors to food security [25]. Unfortunately, many did not report eating these types of wild foods. It has become customary to buy food from markets, and many are unaware of the nutritional benefits found in these foods [25,38]. Select literature regarding food security notes that wild food is consumed as a form of food security in Sri Lanka [25,56]. Research shows that wild food such as fruits, leafy vegetables, mushrooms, tubers and honey increase dietary diversity and greater micronutrient consumption among the rural Sri Lankan communities resulting in improved food and nutrition security [25]. Local edible foods are rich in nutrients, and wild fruit, nuts, seeds and vegetables are good sources of vitamin A and fibre [41].

Urban slum women mainly consume food from the market. Most have low diet diversity due to food prices and low-nutrient food consumption, which is often not prepared by the women themselves. Many of them said that preparing food at home is more expensive than just buying food. People of these areas can easily be accessible for cheap, unhealthy foods (fats, especially foods of animal origin, fast foods (low in fibre and vitamins), salty and oily foods (patties, rolls)). Commonly bought foods are “*Kottu*” fried rice, “*rotie*”, bread, “hoppers”, “string hoppers”. We observed that this food is not nutritious (with the taste of monosodium glutamate (MSG)) and many households are or have switched to similar cheap and low nutritious foods.

The study sample of WRA showed imbalanced macronutrient intake, including a high mean intake of carbohydrate and low mean intake of fat and protein. This means 96% of women met or exceeded the daily recommended carbohydrate intake, while about 83% of women could not reach daily recommended protein intake. High carbohydrate intake can lead to weight gain, poor metabolic health, increased risk of heart disease and type 2 diabetes. On the other hand, white rice is the primary energy source for marginalised Sri Lankan women. Low dietary diversity and grossly inadequate micronutrient intakes have been associated with white rice consumption [69]. Most of the women in the rural study area were eating white rice three times a day. The rice portions are often large and consumed with a small portion of curries (vegetables or meat or fish). While rice is a protein source containing various vitamins (vitamin B, thiamine and niacin) and minerals (zinc and phosphorous), many of are lost during the polishing and milling processes of white rice production. White rice still provides some micronutrients such as riboflavin, folate and niacin, but unprocessed, brown rice has more nutritional benefits [69]. According to the results, monotonous diets were rice-based with a small portion of vegetables, and seldom consumed fruits. As one study in Bangladesh reported, rice is a staple food that contributes to low micronutrients [70]. Additionally, monotonous diets lack essential micronutrients and contribute to malnutrition. Studies by Kennedy et al. [71], Hamlin et al. [72], Chakona et al. [41] reported similar findings.

The study found that women in urban slums had limited micronutrient intake. Nuts, seeds and pulses contain protein, vitamin B, unsaturated fatty acids, fibre and minerals, which have unique health benefits and are rarely consumed by people in urban slums. On the other hand, women in rural areas consumed pulses and beans, nuts and seeds and other vegetables, along with a limited intake of dairy, meat, poultry, fish and eggs. Protein and calcium-rich foods are rarely consumed in this sample. The MDD-W was lower for five food groups consumed by women in urban areas than in rural areas. Previous studies have shown that people of urban areas consume more diverse foods than the rural areas, but the present results reject this finding [73].

### 4.2. Dietary Diversity and Sociodemographic Factors

Several socioeconomic and demographic factors are significantly associated with MDD-W. One such factor is women’s education. A woman with higher education is more likely to be economically independent. It has been well-documented that financial independence has a positive impact on women’s nutrition [74]. A significant positive association was found between maternal education and dietary diversity [75]. This research is comparable to previous studies in Bangladesh and Vietnam. Comparison, along with the urban-rural context showed that the level of food security was higher in rural areas where women with high MDD-W consumed nut and seed, green leafy vegetables, fruits. Savy et al. [76] showed that dietary diversity was related to socioeconomic status. The results show that rural women are better off than their urban counterparts as agriculture is their main source of income and food is easily available in rural areas and often the only limitation is financial access. Therefore, vulnerable people are more likely to have suffered from food shortfalls. Women in urban slums were more food insecure due to high levels of poverty, with a high percentage of the population living in extreme poverty in urban areas. Households living in poverty consume unhealthy food and change their consumption patterns to suit their income. Additionally, urban households have limited access to land (many of the lands are occupied illegally), which can cause them to be more vulnerable to food insecurity than rural households with land access. Dietary diversity was strongly correlated with access to the use of land in this study. A household engaged with the land and its products improved the quality of diets and helped increase food security in low-income households.

### 4.3. Dietary Diversity and Micronutrient Deficiencies

All micronutrients are essential for the proper functioning of the human body. They can act as antioxidants, which may protect against cell damage associated with specific diseases [77]. They are important for a healthy digestive system, and they play an important role in shaping the gut microbiota [78,79]. This research found that the number of daily dietary intake was not sufficient. Other vitamins and minerals such as iron, fat, folate, zinc, thiamine, riboflavin, niacin, and vitamins A, B6, B12, C intakes were less than halfway met. The study results revealed that 72.5% of women did not consume the daily recommended protein intakes. In this sample, 82% of women did not meet daily recommended vitamin A intakes, and more than half did not meet daily recommended riboflavin and vitamin C intakes. Also, this sample results show a high percentage of reproductive-age women are high risk for folate deficiencies. Therefore, these results show that the study area has a high-level risk of micronutrient deficiencies.

Although most women had unhealthy nutritional status, the results revealed that 33.5% of the women involved had a healthy weight. This sample had 43.5% of underweight women, about 19% overweight women and 3.8% obese. Most of the women in study sample were undernutrition. However, the study results demonstrated that rural women’s nutrition status was better than urban women. Nevertheless, this study shows that most of the reproductive-age women in the study area have nutritional problems, and unhealthy diets can have direct or indirect effects on living standards and health [80]. Moreover, some studies have shown that low-income families’ food insecurity is significantly linked to a higher percentage of diabetes [81,82,83]. Some studies have found that nutrition is essential for food security [84,85]. This means that any person suffering from malnutrition or inadequate micro-nutrients can be identified as food unsafe. These shortcomings underscore the importance of knowing household dynamics and individual levels of food security [86].

### 4.4. Diversity of Food Varieties, Agro-Biodiversity, Consumption Patterns, and Health

Reproductive age women are generally at health risk from malnutrition, micronutrient deficiencies during the pregnancy. In the reproductive age, food and nutritional insecurity has been connected with outcomes of poor pregnancy, including low birth weight and gestational diabetes [87,88]. Women were found to be consuming a high amount of carbohydrates while at high-risk for nutritional problems such as diabetics. There was a significant positive relationship between self-reported health status and dietary diversity. In urban areas, women suffer from nutrition-related health problems more than in rural areas. Some studies have shown that dietary diversity is positively associated with nutritional adequacy [89] and also associated with decreased chronic diseases [90], type 2 diabetes [91] and several types of cancer [92,93,94].

This study also showed that indigenous fruits and vegetables were not so popular in both areas, helping to understand the diversity of food and food transition. WRA were not consuming the traditional variety of foods such as healthier wild plants. The notable point is that dairy consumption was low, and women consumed only milk powder. In ancient times people in the area drank goat milk as therapeutic food for allergies and asthma. In these rural areas, people were accustomed to *Mee* oil and sesame oil (see Table 3), but the study revealed that women use coconut oil, vegetable oil and palm oil. In Sri Lanka, there have been several regime changes over the past 400 years and up to now. The process of these changes has accumulated over a substantial period [25]. Food commercialisation, which may impact changing attitudes and dietary patterns, has also disregarded these traditional food sources, especially in agriculture development [95]. This has been evident in Africa, where a decreased agricultural biodiversity has led to a decrease in the variety of food plants grown by household due to agriculture commercialisation [96]. However, when and if available, these conventional varieties can inexpensively increase food security and nutrition security among marginalised societies.

The financial situation plays a major part in obtaining the correct variety of seeds to be planted in the rural areas, fertilisers and protection/safeguarding from aggressors and crop destruction need to be costed to ensure there is sufficient food. Dietary diversity, founded on diverse farming systems, delivers better nutrition and good health, with additional benefits for human productivity and livelihoods. This study proves that agriculture diversity will also be essential to a healthy, sustainable food system, and secure food production. Accordingly, the evidence base for the role of biodiversity in food and nutrition security is growing. For example, a significant positive relationship was found between crop diversity and dietary diversity [97]. The diversification of agricultural production towards fruits, vegetables and aquaculture was seen to improve diet diversity and the intake of specific nutrients [98]. However, this result shows that the biodiversity of plants is vital for humanity’s capacity to meet sustainability challenges. Therefore, to improve food security, the rigorous integration of plant, environmental, social and health is required and should be integrated into policymaking.

All participants’ food preparation methods included long cooking times. However, in the past, different foods had different preparation methods and preservation [25]. Food processing can alter the nutrient quality of foods [99,100] and may have an impact on nutrition problems in these areas. Traditional household food processing and preparation methods can enhance the bioavailability of micronutrients [100] and may be a further topic for investigation. Results show that many women frequently skipped breakfast but fasting for short periods at certain times is not advisable for women of reproductive age. These findings raise a significant concern because of the possible negative impacts their already poor diets may have on maternal health and pregnancy and birth outcomes [101]. The government should pay extra cost for food security and nutrition policies through nutrition supplement for maternal health due to low dietary diversity and inadequate nutrition [102]. The findings also highlight the need to incorporate nutrition interventions that address both food insecurity problems and limited knowledge regarding healthy diets during reproductive age, as recommended by WHO. By providing the cut-off point of five food groups, the MDD-W is a valuable tool to identify and characterise populations with a higher risk of inadequate nutrient intake. Nevertheless, it is important to continue investigating the composition of diets by analysing individual food groups’ consumption. This result will prove the identification of ignored food groups whose production should be promoted to achieve greater diversity. This information is necessitated to assist in designing efficient interventions to improve diet quality. A decreased dependence on self-production and agrobiodiversity, purchasing food at markets, low-income, lack of suitable land, less knowledge about food and nutrition, and reduced diversity of local varieties are the main reasons for food insecurity. Accordingly, food security programs should concentrate on developing among rural and urban marginalised communities. There is a need to enhance the diet and food diversity for women of reproductive age in marginalised areas by training production through home gardening. Consuming a diverse diet is difficult for most Sri Lankan people due to high poverty levels, high unemployment rates, abandonment of agriculture, and increasing food prices. It has been reported that price increases on whole food that contains high nutritional values have an influence on food consumption among vulnerable societies. As a result, many families are switching to cheaper and low nutritious foods that prevent hunger but affect the quality of the food. Households may also decrease the diversity of diets in response to the frequency of meals and decreasing portion size.

Based on these research results, there is a need to enhance traditional food culture and farming systems. Our results enable policymakers or competent authorities to develop appropriate nutrition programs for vulnerable people in marginalised areas and to strengthen sustainable food and nutrition systems in Sri Lanka. Further studies should be conducted to continue investigating the composition of diets by analysing individual food groups’ consumption, micronutrition deficiencies and dietary diversity. In the preliminary analyses presented in this article, list-based and open methods were applied to avoid the concern that both MDD-W and nutrient intake came from the same 24 h recall. Repeating the analyses using MDD-W derived from the 24 h dietary intake data did not change our finding that the MDD-W performed poorly in identifying individuals portion size with micronutrient intake among reproductive-age women. The recall of food group intake through the list-based and open methods was done on the same day as the multiple-pass 24 h recall. The advantage of the MDD-W derived from the list-based method was similar to the 24 h open recall of the data.

There are several strengths connected with this study. One of the key forces is the representational nature of the data used in the analysis. This means that the findings of the study cannot be generalised to all reproductive women in Sri Lanka. To the best of our knowledge, this is the first study in Sri Lanka using nationally representative data to investigate MDD-W in marginalised communities. There are several limitations to this study. This model indicates the advantages effects of nutrition on health promotion, disease management and risk reduction. The data determined from the data of a cross-sectional survey did not allow us to determine causality. If both areas were significantly larger, and the sample size was relatively small, this may be have affected accuracy and reliability. Therefore, the sample size 400 was selected, indicating a confidence gap of ±2.5% (total = 5%) and the testers were trained to check the data. This study examined two different marginalised communities. Therefore, a marginalised community is the people in Sri Lankan society who are at risk from existing nutritional, health and other social problems. Women of reproductive age are a good indicator of the general well-being of the community. The study examined two different marginalised areas with WRA. These results could not be generalised to other settings but should be restricted to WRA in marginalised areas.

In addition, the evaluation of this study was self-reporting in WRA. The BMI symbolised the relationship between MDD-W and demographic factors of nutritional status. This research did not investigate the nutritional status and relied upon self-reported health conditions without health examinations. Additionally, the study estimated only one 24 h recall per woman dietary intake. Although this is appropriate to measure populations’ mean intakes, it is inadequate to capture the day-to-day variation in intakes. Therefore, for further investigation, it is recommended to utilise a different kind of dietary questionnaire to collect and capture intakes, such as a food frequency questionnaire or 7-day dietary recall [103]. Using a dichotomous indicator could be less sensitive in identifying the relation with potential determinants compared with a continuous indicator such as WDDS. Still, our findings invalidated this hypothesis for our sample. WDDS indicator was to be used as a continuous variable (ranging from 0 to 9 food groups consumed) and averaged to generate a mean value for populations. It did not allow us to assess the percentages of the population with low or adequate dietary diversity. WDDS failed to identify a single, universal cut-off point that would accurately classify women into those with low dietary diversity and those with minimum dietary diversity across the different contexts. For this reason, FAO developed the minimum dietary diversity in women (MDD-W) indicators in 2014. When the MDD-W indicator is at the population level, the indicator is a good proxy for predicting micronutrition, but it does not perform well in individual women. By providing the cut-off point of five food groups, the MDD-W is a valuable tool to identify and characterise populations at greater risk of inadequate nutrition intakes.

## 5. Conclusions

In conclusion, this study observed that women of reproductive age had poor diets with imbalanced macronutrients and alarmingly low intakes of some important micronutrients. Study results showed that women in urban slums reported higher food insecurity than women in rural areas. Additionally, monotonous diets were rice-based, with little vegetables and rarely consumed fruits. This sample did not find any consumption of a traditional variety of foods, and agrobiodiversity is decreasing. Sri Lankan food patterns are changing toward low dietary diversity and low-quality diets such as high fat and carbohydrates. There is a need to improve traditional food culture, farming methods and nutrition education. Study results proved that dietary diversity and food security are correlated with income and level of education, but food expenditure, household size and main occupation were not. Furthermore, double-burden malnutrition is related to dietary diversity. Reproductive age women’s diets revealed a higher MDD-W within the rural areas, and the lower MDD-W were seen in the urban areas. As briefly noted in the research study, imbalanced nutrition will impact future generation’s nutrition and health status. Marginalised society is at high risk for nutritional deficiencies, heart disease, diabetes, cancer, kidney disease, high blood pressure and eyesight problems in reproductive women, all associated with dietary diversity. This research finding helped to understand the diversity of food and food transition and revealed that indigenous fruits and vegetables were not so popular in both areas. We conclude that there is a need to enhance nutrition education about diet diversity and food security among reproductive women.

Further studies can investigate the strategic nutrient intake and micronutrition problems in these areas among women and children. Accordingly, every country needs strong governance to address the nutrition challenge successfully. The study demonstrated that food and nutrition policies change due to changes in political regimes. Local governments play a role in increasing access to healthy foods and reducing access to unhealthy food. According to our finding and reflected in literature, to date, there is more access to enhancing strategies than those that might reduce access to unhealthy food in marginalised societies. However, the local government should also focus on strategies that reduce access to unhealthy foods. Local governments can have a strong and direct impact on people’s health and well-being.

## Figures and Tables

**Table 1:** Demographic and socioeconomic characteristics of women in the reproductive age group (*n* = 400).

Characteristic *n* = 400	Rural (%)	Urban (%)	Total Value (%)
Age (years)			
18–25	72 (36)	55 (27.5)	137 (31.8)
26–35	72 (36)	64 (32)	136 (34)
36–49	56 (28)	81 (40.5)	137 (34.3)
Monthly income (Rs)			
1–5000	32 (16)	4 (2)	37 (9)
5001–10,000	5 (3.5)	40 (20)	44 (36.2)
10,001–20,000	67 (33.5)	82 (41)	149 (37.3)
20,001–30,000	45 (22.5)	51 (25.5)	196 (24)
30,001–40,000	35 (17.5)	17 (8.5)	52 (13)
40,001–50,000	15 (7.5)	4 (2)	19 (4.8)
50,000<	1 (0.5)	2 (1)	3 (0.8)
Food Expenditure (Rs)			
1–5000	100 (50)	22 (11)	122 (30.5)
5001–10,000	14 (7)	70 (35)	84 (21)
10,001–20,000	80(40)	80 (40)	160 (40)
20,001–30,000	6 (3)	24 (12)	30 (7.5)
30,001–40,000	-	4 (2)	4 (1)
Family Size *			
Small family	117 (58.5)	84 (42)	201 (50.3)
Large family	83 (41.5)	116 (58)	199 (49.8)
Main Occupation			
Unemployed	123 (61.5)	119 (59.5)	242 (60.5)
Agriculture	68 (34)	-	68 (17)
Labour job ***	5 (2.5)	74 (37)	79 (19.8)
Government Job	4 (2.0)	7 (3.5)	11 (2.8)
BMI level			
Underweight	74 (37)	100 (50)	174 (43.5)
Normal weight	85 (42.5)	49 (24.5)	134 (33.5)
Overweight	37 (18.5)	40 (20)	77 (19.3)
Obese	4 (2)	11 (5.5)	15 (3.8)
Education level			
Non-educated	16 (8)	21 (10.5)	37 (9.3)
Primary	86 (43)	148 (74.)	234 (58.5)
Secondary	95 (47.5)	30 (15)	125 (31.3)
Junior secondary **	55 (27.5)	28 (14)	83 (20.75)
Senior secondary	40 (20)	2 (1)	42 (10.5)
Higher	3 (1.5)	1 (0.5)	4 (1)
Sources of the food			
Own production	141 (70.5)	-	141 (35.5)
Purchase	25 (12.5)	197 (98.5)	222 (55.5)
Collected or donated	33 (16.5)	3 (1.5)	36 (9)

* According to the concept of family planning in Sri Lanka: A small family is a father, a mother and two children, and a large family is a father, a mother, and three or more children [51], *** They work as casual workers. ** Up to 9th grade (age 14): Under Sri Lankan law, all children must go to school up to 9th grade.

**Table 2:** Types of foods consumed in WRA in marginalised areas.

Food Groups	Correlation	*p*-Value	Urban Area		Rural Area	
			Mean ± S.D.	Food Items	Mean ± S.D.	Food Items
01. Starchy staple foods (grains, white roots and tubers plantations)	-	-	1.00 ± 0.000	Rice (*Oryza sativa*), wheat (Triticum aestivum L.), potatoes *(Soanum tuberosum* L.)	1.00 ± 0.000	Rice (*Oryza sativa*), wheat (*Triticum aestivum* L.),jackfruit (Artocarpus heterophyllus),*Katu ala* (*Dioscoreapentaphylla*), breadfruit (*Artocarpus*), cassava (*Manihot esculentum*), sweet potatoes *(Ipomoea batatas*), *Kiri ala* (*Xanthosomasagittifolium*), lotus root (*Nelumbo nucifera*), bananas/unripe (*Musa*), potatoes (*Solanum tuberosum* L.)
02. Pluses, beans peas and lentils	−0.336 **	0.000 **	1.35 ± 0.478	Long bean *(Vigna subterranean*), bean (*Vigna angularis*), chickpea (*Cicer arietinum*), lentil/*dal* (*disambiguation*). *Soya* (textured soy protein/TSP)	1.08 ± 0.264	Mung bean (Vigna radiata), cowpea (*Vigna unguiculata*), long bean *(Vigna subterranean*), bean (*Vigna angularis*), chickpea *(Cicer arietinum*), winged bean (*Psophocarpus tetragonolobus*), lentil/ *dal* (*disambiguation*). Soya (textured soy protein/TSP)
03. Nuts and seeds	−0.840 **	0.000 **	1.42 ± 0.272	Peanut (*Arachis hypogaea*), coconut palm (*Cocos nucifera*)	1.08 ± 0.272	Peanut (*Arachis hypogaea*), cashew (*Anacardium occidentale*), coconut palm (*Cocos nucifera*)
04. Dairy product (e.g., milk, yoghurt and cheese	0.292 **	0.000 **	1.62 ± 0.494	Milk powder, yoghurt, curd	1.77 ± 0.457	Milk powder
05. Meat (all meat fish, chicken and liver or organ meat)	0.537 **	0.000 **	1.16 ± 0.363	Chicken, red meat, fresh or dried seafood, canned fish (sardines)	1.69 ± 0.466	Chicken, fresh or dried fish (seafood or freshwater fish/tank fish)
06. Eggs	0.130 **	0.009 **	1.77 ± 0.422	Chicken eggs	1.87 ± 0.337	Chicken eggs
07. Dark green leafy vegetables	0.492 **	0.000 **	1.85 ± 0.358	*Sarana* (*Trianthema portulacastrum*), *Kankung* (*Ipomoea aquatica*) *Gotukola* (*Centella asiatica*), *Mukunuvanna* *(Alternanthera sessilis*)	1.37 ± 0.487	*Kankung* (*Ipomoea aquatica*), *Gotukola* (*Centella asiatica*), *Mukunuvanna* (*Alternanthera sessilis*), manioc leaves (*Maniot esculenta*), *Kathurumurunnga (Sesbania grandiflora*), pumpkin leaves (*cucurbita maxima*), *Japan batu* (*Sauropus androgynus*), *Thebu* (*Costus speciosus*), passionfruit leaves (*Passiflora edulis*)
08. Vitamin-A rich fruits and vegetables	0.272 **	0.000 **	1.96 ± 1.146	Carrot *(Daucus carota*), pumpkin *(Cucurbita pepo*), papaya (*Carica papaya*)	1.65 ± 1.132	Carrot (*Daucus carota*), pumpkin (*Cucurbita pepo*), papaya (*Carica* *papaya*), mango (*Mangifera indica*)
09. Other vegetables	−0.476 **	0.000 **	1.60 ± 0.491	Radish (*Raphanus sativus*), aubergine (*Solanum melongena*), bitter gourd *(Momordica charantia*)*,* ridge gourd (*Luffa),*	1.18 ± 0–488	Radish (*Raphanus sativus*), aubergine (*Solanum melongena*), bitter gourd (*Momordica charantia*), ridge gourd (*Luffa*), snake cucumber/kekiri (*Cucumis melo*), tomato (*Solanum Lycopersicum*), plantain flower, *ambarella* (*Spondias Dulcis*), wild eggplant/*Thibbatu* (*Solanum torvum*)
10. Other fruits	−0.067	0.180	1.82 ± 0.389	Banana (*Musa paradisiaca* L.), apple (*Malus pumilamill*.)	1.39 ± 0.488	Banana (*Musa paradisiaca L*.), apple (*Malus pumila Mill.*)

** Statistical significance *p* < 0.01 (two-tailed), S.D: standard deviation.

**Table 3:** Commonly used traditional food varieties in rural areas.

Vernacular Name	Crop Group	Botanical Name	Nutritional and Therapeutic Value
Annona/*Atha*	Fruits	*Annona muricate/Annona reticulata*	The flowers and ripe fruits were used to disinfect the body. Unripe fruits helped to prevent diarrhoea. Fresh leaves were applied to the stomach of children with indigestion. It contains high antioxidants, vitamin C, manganese, thiamine, vitamin B, iron, phosphorus and potassium.
Wood Apple/*Divul*	Fruits	*Feronia Limonia* L.	The unripe fruits were used for chronic diarrhoea and dysentery. It contains protein, carbohydrates, iron, fat, calcium, vitamin C, and B.
Bel fruit/*Beli*	Fruits	*Aegle Marmelos* L.	This fruit was used for fever, hypochondria, melancholy, heart palpitations, diarrhoea and gastric disorders. The leaves were used for jaundice and anasarca. Dried leaves, flowers and fruit petals were used to cool the body as well as in traditional medicine. It contains Beta-carotene, protein, riboflavin, niacin, carotene, calcium, potassium, fibre and healthy fats.
Ash Pumpkin/*Puhul*	Vegetables	*Benincasa hispida*	The fruit contains a fixed oil, starch, resin, proteins and vitamins B and C. It is used for insanity, epilepsy and other nervous diseases. The cortical part of the fruit is given to diabetics.
Horse purslane/*Heen sarana*	Green leafy Vegetables	*Trianthema portulacastrum*	This plant was used for jaundice, anaemia, asthma, liver disorders, dysuria, constipation, swelling. The plant contains protein, energy, fat, calcium, phosphorus, iron, vitamin A.
*Tel kola*	Green leafy Vegetables	*Ipomoea*	Internally, this plant helps as a cardiac, stomachic, expectorant and diuretic and is useful for chronic dyspepsia, bronchitis and revel and hepatic dropsy. The leaves contain vitamin A and iron.
Black gram/*Udu*	Grains	*Phaseolus mungo*	The seed contains moisture, energy, proteins, fats, carbohydrates, calcium, phosphorus, carotene, thiamine, riboflavin and niacin This was used in the treatment of fever, piles, cough and liver diseases.
Tamarind/ *Siyabala*	Condiments and Seasonings	*Tamarindus indica*	The fruits contain energy, protein, moisture, fats calcium, carotene, riboflavin, and niacin. It was used in the treatment of fever, piles, cough and liver diseases.
Drumstick/*Murunga*	Vegetables	*Moringa oleifera*	The fruits contain energy, iron, moisture, protein, fats, carbohydrates, calcium, phosphorus, carotene, thiamine, riboflavin, niacin and vitamins C and B. It was used for insanity, epilepsy and other nervous diseases. The cortical parts of the fruit are given for diabetes. Leaves and antidote bark of the tree are used in food preparation.
Palmyra Palm/*Thal,*	Fruits	*Borassus flabellifer*	This fruit was used to prepare for toddy, jaggery and vinegar. Young nut water was consumed. The fruit contains vitamins B and C, iron, zinc, potassium, calcium, phosphorus, riboflavin and thiamine. This fruit was used to prevent diabetes.
*Maa-dan*	Fruits	*Syzygium caryophyllatum*	Fruits contain moisture, energy, protein, fat, carbohydrates, calcium, phosphorus, carotene, thymine, riboflavin, niacin, iron and vitamin C.
*Kekatiya* regarded as threatened species (IUCN red list)	Vegetables	*Aponogeton crispus*	This plant was eaten as a vegetable and was used for the burning sensation of the body, heart disease, injury, excessive thirst and vomiting.
Wild Asparagus/*Haathavariya*	Beverages	*Asparagus racemosus*	This plant was used traditional medicine to treat ailments such as urinary difficulties, menopausal symptoms, to increase lactation, and reduce the risk of cancer.
Finger millet/*Kurakkan*	Grains	*Eleusine coracana*	This cereal contains protein, fibre, iron and is fortified with vitamins and minerals. It has a wide range of benefits and helps to reduce weight, cholesterol, to control diabetes and to cool the body. This cereal improves digestion and makes bones stronger.
Honey tree/*Mee*	Oil	*Madhuca loggifolia*	Flowers, seeds, and seed oils had traditional medicinal value. *Mee* oil was used not only as a massage oil but also as cooking oil. The oil is considered a good remedy for swelling, broken bones, itching and snakebites. It is given internally to treat diabetes and chronic tonsillitis.
Jack Seeds	Nuts and seeds	*Arto Carpus heterophyllus*	It has a high content of vitamin A, vitamin C, niacin, calcium, thiamine, riboflavin, potassium, iron, magnesium. Jack seeds are a great source of iron. It helps to prevent mental stress and skin diseases.
Lotus Seeds	Nuts and seeds	*Nelumbo nucifera*	These seeds are low in cholesterol and saturated fat. Lotus seeds are good for the heart and have high magnesium, potassium, protein and phosphorus content.
*Kohila*	Green Leaf Vegetables	*Lasia Spinosa*	Kohila is used as a vegetable. There are several varieties kohila such as Kiri kohila, well kohila, guru kohila, Kalu kohila and goda kohila. The tubers, roots and leaves are used as medicine. It contains lots of fibre, calcium and vitamin C.

Sources: [25,52,53,54,55,56].

**Table 4:** Minimum dietary diversity score of women of reproductive age (MDD-W).

Areas	Mean ± S.D.	Women of Reproductive Age (%)	
		<5 Food Groups	≥5 Food Groups
Urban	1.16 ± 0.368	84	16
Rural	1.58 ± 0.496	42.5	57.5
Total	1.37 ± 0.483	63.25	36.75

S.D: standard deviation.

**Table 5:** Associations between socioeconomic indicators and MDD-W.

Indicators	Spearman Correlations	*p*-Value	Mean ± S.D.	β	S.E.	t
Area	0.430 **	0.000 **	1.50 ± 0.501	0.444	0.047	9.023
Age	−0.042	0.398	2.03 ± 0.813	−0.010	0.028	−0.210
Monthly income	0.148 **	0.003 **	2.47 ± 1.137	0.040	0.025	0.673
Food expenditure	−0.033	0.505	1.58 ± 0.674	−0.019	0.042	−0.321
Family size	−0.012	0.815	1.50 ± 0.501	0.058	0.046	1.219
Sources of food	−0.136 **	0.007 **	2.03 ± 0.393	−0.143	0.057	−3.086
Marital status	0.043	0.394	0.35 ± 0.823	−0.001	0.028	−0.015
BMI level	0.147 **	0.003 **	1.83 ± 0.667	0.057	0.026	1.255
Main occupation	0.019	0.704	0.065 ± 0.889	−0.028	0.031	−0.532
Education level	−0.144 **	0.004 **	1.24 ± 0.623	0.037	0.000	0.010
Health condition	−0.203 **	0.000 **	1.76 ± 2.101	−0.136	0.010	−3.415

Abbreviation: β—beta weights (standardised beta coefficient), SE—std. Error ** statistical significance *p* < 0.01 (two-tailed), S.D: standard deviation.

**Table 6:** Self-reported health condition.

Type of Conditions	Urban *n* (%)	Rural *n* (%)	Total *n* (%)
Cancer	10 (5)	3 (1.5)	13 (3.25)
Diabetes	23 (11.5)	3 (1.5)	13 (3.25)
Heart disease	2 (1)	-	2 (0.5)
High blood pressure	12 (6)	7 (3.5)	19 (4.5)
Chronic kidney diseases	-	5 (2.5)	5 (1.25)
Thyroid related diseases	5 (2.5)	1 (0.5)	6 (1.5)
Eyesight problems	5 (2.5)	6 (3)	11 (2.75)
Other diseases	47(23.5)	20 (10)	37 (11.8)

**Table 7:** Selected macro and micro vitamins and minerals intake.

Selected Vitamin and Mineral Intake	SD	EAR ^1^	Mean Intake	*p*-Value
Carbohydrate/g	0.196	130	300	0.126
Fat/g	0.809	69	58	0.000
Protein /g	1.246	46	23.4	0.000
Iron /mg	0.805	8.1	7.2	0.000
Vitamin A/μg	1.227	500	180	0.000
Calcium/mg	1.230	800	220	0.000
Zinc /mg	0.479	6.8	1.2	0.021
Thiamine/mg	0.496	0.9	0.98	0.076
Riboflavin/mg	2.182	0.9	0.99	0.082
Folate/μg	0.906	320	48.6	0.000
Niacin /mg	0.823	11	11.6	0.671
Vitamin B6/mg	0.818	1.1	0.96	0.000
Vitamin B12/μg	0.808	2.0	0.86	0.000
Vitamin C/mg	1.158	60	24.4	0.000
Water/L	0.912	2.7	2.0	0.000

^1^ EAR: estimated average requirements per day [45,46]. SD: standard deviation.
